# Early and Late Influenza Vaccine Effectiveness in South Korea During the 2023–2024 Season

**DOI:** 10.3390/vaccines13020197

**Published:** 2025-02-17

**Authors:** Yu Jung Choi, Joon Young Song, Seong-Heon Wie, Jacob Lee, Jin-Soo Lee, Hye Won Jeong, Joong Sik Eom, Jang Wook Sohn, Won Suk Choi, Eliel Nham, Jin Gu Yoon, Ji Yun Noh, Hee Jin Cheong, Woo Joo Kim

**Affiliations:** 1 Division of Infectious Diseases, Department of Internal Medicine, Korea University College of Medicine, Seoul 02841, Republic of Korea; yujungchoi@kumc.or.kr (Y.J.C.);; 2 Vaccine Innovation Center-KU Medicine (VIC-K), Seoul 02841, Republic of Korea; 3 Division of Infectious Diseases, Department of Internal Medicine, St. Vincent’s Hospital, College of Medicine, The Catholic University of Korea, Suwon 16247, Republic of Korea; 4 Division of Infectious Diseases, Department of Internal Medicine, Kangnam Sacred Heart Hospital, Hallym University College of Medicine, Seoul 06351, Republic of Korea; 5 Division of Infectious Diseases, Department of Internal Medicine, Inha University School of Medicine, Incheon 22332, Republic of Korea; 6 Department of Internal Medicine, Chungbuk National University College of Medicine, Cheongju 28644, Republic of Korea; 7 Division of Infectious Diseases, Department of Internal Medicine, Gil Medical Center, Gachon University College of Medicine, Incheon 21999, Republic of Korea

**Keywords:** influenza, influenza vaccine, vaccine effectiveness

## Abstract

Background: During the 2023–2024 season, the influenza epidemic in South Korea peaked earlier, and the influenza vaccination rate among individuals aged ≥ 65 was high (82.2%). However, data on real-world vaccine effectiveness against influenza are lacking. Methods: From November 2023 to April 2024, we conducted a multicenter retrospective case–control study on adult patients aged ≥ 18 years who presented with influenza-like illness at seven medical centers as a part of a hospital-based influenza morbidity and mortality surveillance (HIMM) program in South Korea. Demographic and clinical data were collected from questionnaire surveys and electronic medical records. Using a test-negative design, we assessed the effectiveness of the 2023–2024 seasonal influenza vaccine, with age, sex, and comorbidities included as covariates. Results: A total of 3390 participants were enrolled through the HIMM system, including 1695 patients with either rapid antigen test (RAT) or real-time reverse-transcription polymerase chain reaction (RT-PCR) positive results and controls matched for age, sex, and months of registration. Among the 1696 influenza-positive patients, 1584 (93.5%) underwent RAT, with 88.9% testing positive for influenza A and 11.1% for influenza B. During the study periods, the overall vaccine effectiveness (VE) was 24.3% (95% confidence interval (CI), 11.5 to 35.2). The VE was insignificant when limited to older adults aged ≥ 65 years (13.5%; 95% CI, −17.9 to 36.6). In the subgroup analysis by subtype, the VE was 19.0% (95% CI, 5.0 to 31.0) for influenza A and 56.3% (95% CI, 35.3 to 70.6) for influenza B. Notably, influenza VE was 20.4% (95% CI, 2.9 to 34.8) in the early period (November to December) but decreased to 12.4% (95% CI, −14.9 to 33.2) in the late period (January to April). Conclusion: During the 2023–2024 season, the influenza vaccine showed a modest effectiveness (24.3%) against laboratory-confirmed influenza, which was particularly higher for influenza B. Because the VE was insignificant in older adults, particularly during the late period, better immunogenic influenza vaccines with longer-lasting protection should be considered.

## 1. Introduction

Influenza is a respiratory disease caused by infection with the influenza virus and is prevalent in the Northern Hemisphere from November to April. According to the Centers for Disease Control and Prevention (CDC), the 2023–2024 seasonal influenza circulation pattern has returned to pre-pandemic norms, showing a closer resemblance to the patterns observed during the two seasons (2020–2021 and 2021–2022) when severe acute respiratory syndrome coronavirus 2 first emerged [1]. Influenza A (H1N1pdm09) was reported as the most prevalent subtype early in the 2023–2024 season, with influenza B or A (H3N2) becoming more prevalent later in the Northern Hemisphere [1,2]. In South Korea, the circulation patterns were similar, with the prevalence of influenza-like illness (ILI) peaking (61.3%) in the second week of December [3].

The disease burden of influenza remains a global challenge. Although the effectiveness of influenza vaccination in preventing infections may be limited, it plays a crucial role in preventing hospitalizations and deaths associated with influenza, thereby reducing the overall burden of the disease [4]. In South Korea, the influenza vaccination rate for the elderly (aged ≥ 65 years) is high (82.5%) [5], but data on real-world vaccine effectiveness (VE) against influenza are lacking. However, there are few reports from Asian countries; various organizations in other countries within the Northern Hemisphere evaluate and report the VE of the influenza vaccine every season [6,7,8,9].

In this study, we aimed to evaluate the real-world influenza VE in the 2023–2024 season and provide valuable resources for enhancing our response to influenza.

## 2. Methods

### 2.1. Study Design and Data Collection

This study was a multicenter retrospective case–control study using a hospital-based influenza surveillance system in South Korea (hospital-based influenza morbidity and mortality [HIMM]). HIMM included eight university hospitals from various regions of Korea. From November 2023 to April 2024, we included adult patients (aged ≥ 18 years) who met the following criteria: (1) presented with ILI at emergency departments or outpatient clinics, (2) were hospitalized with laboratory-confirmed influenza, regardless of symptoms, or (3) were hospitalized with symptoms consistent with ILI. Individuals less than 18 years of age were excluded. ILI was defined as a fever of ≥38 °C accompanied by one or more respiratory symptoms, such as cough, sore throat, or rhinorrhea, occurring within the past 7 days.

We conducted an influenza rapid antigen test (RAT) or real-time reverse-transcription polymerase chain reaction (RT-PCR) according to the standard protocols. RAT is performed more frequently in Korea than PCR tests for cost and rapidity considerations. Because RAT is known to be less sensitive, we limited this test to patients who underwent testing within 48 h of symptom onset. The RAT positive percent agreement (PPA) was 55.6% (69/[69 + 55]), and the negative percent agreement (NPA) was 94.0% (173/[173 + 11]). For RT-PCR, the PPA was 86.3% (69/[69 + 11]), and the NPA was 75.9% (173/[173 + 55]). The details of this are provided in Supplementary Table S1. We defined influenza-positive as testing positive for influenza by RAT or RT-PCR. The control group was defined as the test-negative group matched for age, sex, and month of enrollment.

Demographic and clinical data were collected through questionnaire surveys and electronic medical records from each center. The history of vaccination was collected through individual questionnaires, medical records, and the national immunization registry of the Korea Disease Control and Prevention Agency (KDCA). We considered vaccinations valid if they were administered at least 14 days prior to the diagnostic testing of influenza.

### 2.2. Statistical Analysis

We assessed the effectiveness of the 2023–2024 seasonal influenza vaccine using a test-negative design, the most commonly used method for evaluating VE. Categorical variables are presented as numbers and percentages. Differences in demographic and clinical characteristics between groups were estimated using Chi-square or Fisher’s exact tests. A logistic regression analysis was used to estimate the relationship between VE and laboratory-confirmed influenza, with age, sex, and comorbidities included as covariates. Similarly, additional logistic regression analyses were conducted to estimate the relationship between VE and influenza-related hospitalizations and ICU admissions. Subgroup analyses were performed by influenza virus subtypes and periods. Statistical analyses were performed using IBM SPSS Software version 25.0 (IBM Corp., New York, NY, USA). A *p* < 0.05 was considered statistically significant.

### 2.3. Ethics Statement

This study was approved by the Institutional Review Board of each participating hospital: Korea University Guro Hospital, 2022GR0360; Korea University Anam Hospital, 2022AN0449; Korea University Ansan Hospital, 2022AS0226; St. Vincent’s Hospital, VC22TIDI0150; Kangnam Sacred Heart Hospital, HKS, 2022-07-016; Inha University Hospital, 2022-07-036; Chungbuk National University Hospital, 2022-08-022; and Gil Medical Center, GAIRB2022-306. It was conducted in accordance with the Code of Ethics of the World Medical Association (Declaration of Helsinki). The requirement for written informed consent was waived due to the retrospective nature of this study.

## 3. Results

### 3.1. Baseline Characteristics of the Study Participants

A total of 3390 participants were enrolled through the HIMM system, including 1695 with either RAT or RT-PCR positive results and test-negative controls matched for age, sex, and months of enrollment with ILI. A total of 3289 (97.0%) participants underwent RAT, with 1408 (42.8%) testing positive for influenza A and 177 (5.4%) for influenza B. A total of 409 participants underwent RT-PCR, with 170 (41.6%) testing positive for influenza A and 10 (2.5%) for influenza B (Supplementary Table S1).

The demographic and clinical characteristics of the study population are shown in Table 1. In each group, 2140 participants were enrolled during the early period (November to December) and 1250 participants during the late period (January to April). Among the participants, 1988 (58.6%) were less than 50 years old, and 435 (25.7%) were 65 years of age or older. Compared to the test-positive cases, individuals in the control group were more likely to have underlying medical conditions, including chronic renal disease, chronic liver disease, solid malignancies, and use of immunosuppressive agents (Table 1). The hospitalization rates were higher in the control group than in the case group (34.0% vs. 22.8%, *p* < 0.001), which may be attributed to differences in underlying medical conditions between the groups. Intensive care unit (ICU) admission and mortality rates showed a similar trend between the groups (5.6% vs. 3.6%, *p* < 0.001; 2.1% vs. 1.3%, *p* < 0.001).

### 3.2. Vaccine Effectiveness (VE) for the 2023–2024 Season

Across the study periods, the overall VE was 24.3% (95% confidence interval [CI], 11.5 to 35.2), which was particularly higher in the younger adult group (31.1%; 95% CI, 15.7 to 43.7). The VE was not statistically significant when the analysis was limited to older adults aged ≥ 65 years (13.5%; 95% CI, −17.9 to 36.6) (Supplementary Table S2). The VE against influenza-related hospitalizations and ICU admissions was 16.5% and 55.2%, respectively, but these results were not statistically significant (Table 2).

**Figure 1:**
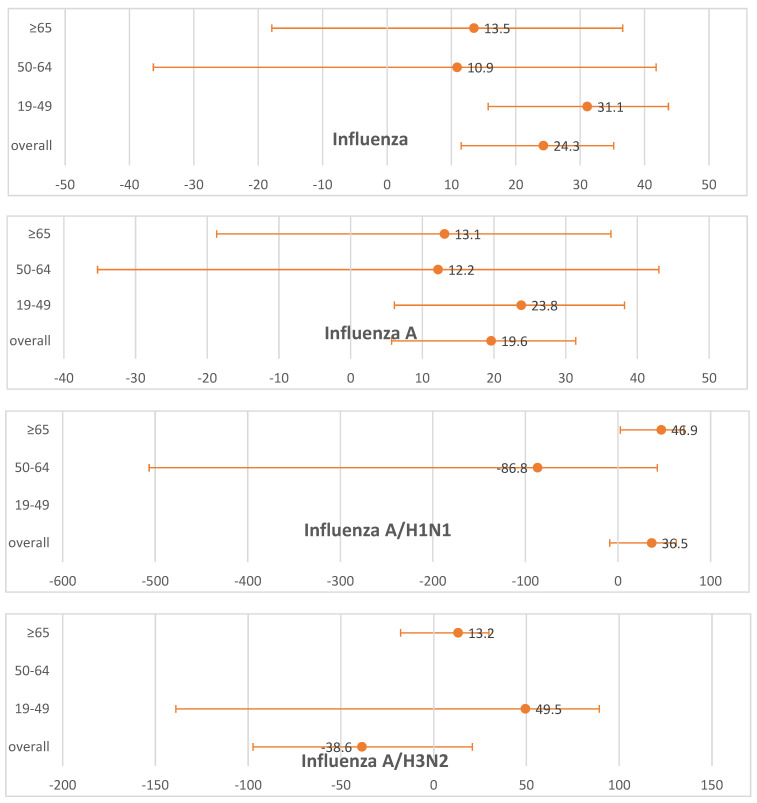

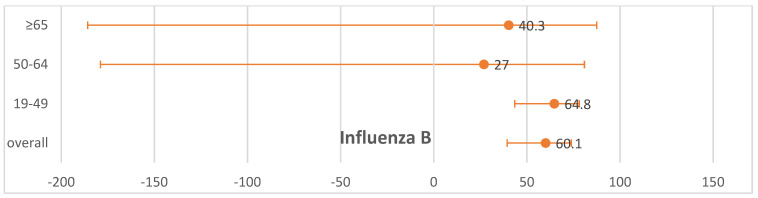
Forest plot of estimated influenza vaccine effectiveness based on subtypes.

In the subgroup analysis by subtypes, the VE was 19.0% (95% CI, 5.0 to 31.0) for influenza A and 56.3% (95% CI, 35.3 to 70.6) for influenza B. A similar pattern was observed for both subtypes, with the VE being significantly lower in older adults than in younger adults, i.e., those under 50 years of age (influenza A, 23.8 vs. 13.1; influenza B, 64.8 vs. 40.3). For the subgroup analysis of H1N1 and H3N2, statistical significance could not be determined due to the small sample sizes. The forest plot for the age-stratified subtype subgroup analysis is presented in Figure 1.

### 3.3. Comparison Between the Early and Late Periods of the 2023–2024 Season

During the early period, 96.8% of cases were confirmed to be influenza A, with 62.6% and 37.4% being H1N1 and H3N2 influenza, respectively. During the late period, the prevalence of H3N2 increased to 86.0%, and that of influenza B reached 23.8% (Supplementary Table S3). According to surveillance data by the KDCA [10], influenza A/H1N1 was more prevalent in the early part of the 2023–2024 season, while the H3N2 and B strains dominated later in the season, which is consistent with our findings.

The overall influenza VE was 21.5% (95% CI, 4.3 to 35.5) during the early period and 28.1% (95% CI, 7.2 to 44.4) during the late period. However, for influenza A, the VE was 20.4% (95% CI, 2.9 to 34.8) in the early period but decreased to 12.4% (95% CI, −14.9 to 33.2) in the late period (Table 3).

## 4. Discussion

In this study, we estimated the influenza VE from November 2023 to April 2024 during the 2023–2024 season. The overall VE was 24.3% (95% CI, 11.5 to 35.2), with 19.6% for influenza A and 60.1% for influenza B. The VE was lower among older adults than among younger individuals. In the subgroup analysis, the VE for influenza A decreased to 12.4% (95% CI, −14.9 to 33.2) in the late season compared with 20.4% (95% CI, 2.9 to 34.8) in the early season.

The matching of the vaccine strains with the circulating strains is the most crucial factor in VE [11]. According to CDC surveillance data, all influenza B viruses with available lineage information were identified as belonging to the Victoria lineage, which is consistent with the vaccine strain [1]. Our study demonstrated a notably high VE for influenza B, with an overall VE of 60.1% (95% CI, 39.4 to 73.8) in the subgroup analysis. The effectiveness was particularly high at 64.8% in young patients less than 50 years of age. These results highlight the importance of matching vaccine strains with circulating strains to improve VE.

A comparison of VE between the early period (within two months post-vaccination) and the late period (three to six months post-vaccination) showed a significant decline in vaccine effectiveness over time. This observation is consistent with previous studies on antibody kinetics, which demonstrate a rapid decline in antibody titers shortly after the initial immune response. According to Song et al., the peak antibody response is achieved within a few weeks of vaccination; a significant reduction in titers is observed within three to six months, potentially contributing to reduced VE in the later period [12]. Similarly, Castilla et al. emphasized that VE tends to decrease over time, particularly in older adults, due to both antibody waning and the natural decline in immune system functionality [13]. These findings suggest that influenza vaccines provide robust initial protection, but effectiveness declines as antibodies wane, highlighting the need for highly immunogenic vaccines such as high-dose, MF59-adjuvanted or recombinant influenza vaccines.

In this study, the overall VE in the 2023–2024 season was lower than that reported in some other countries during their interim analyses, which aligns with the findings from our own interim analysis previously published [14]. While Canada reported a notably high overall VE exceeding 60%, a study including six European countries found that the VE against influenza A ranged from 38% to 51% [15,16]. Interim estimates in the United States indicated that the 2023–2024 seasonal influenza VE ranged from 33% to 67%, depending on the age group and setting [17]. VE can vary significantly across regions, reflecting differences in circulating influenza virus strains in different geographic areas and underscoring the importance of regional surveillance. There are regional differences in the circulating subclades of influenza viruses. For influenza A(H1N1) pdm09, the 5a.2a.1 and 5a.2a clades were predominant during the 2023–2024 season. In Europe, 44% of the strains belonged to the vaccine-matched clade 5a.2a.1, while in Canada, 51% were consistent with the vaccine clade 5a.2a.1, contributing to the higher VE observed in Canada [16,18]. Similarly, 75.6% of cases in the United States were associated with the clade 5a.2a.1 in the full analysis [19]. Unfortunately, laboratory surveillance results are not yet available for South Korea.

The VE was observed to be lower in older adults than in younger individuals across all influenza subtypes. It is a well-established fact that vaccine immunogenicity tends to decline with age, primarily due to immunosenescence, the gradual deterioration of the immune system associated with aging [20]. In Korea, the inactivated quadrivalent influenza vaccine is provided free of charge to children aged 6 months to 13 years, pregnant women, and older adults aged ≥ 65 [21]. The CDC’s Advisory Committee on Immunization Practices (ACIP) recommends that adults aged ≥ 65 should preferentially receive any one of the highly immunogenic vaccines, including the high-dose influenza vaccine, MF59-adjuvanted influenza vaccine, or recombinant influenza vaccine [22]. Recent studies have shown that high-dose or adjuvanted influenza vaccines induce more effective immune responses in older adults than standard-dose vaccines [23,24,25]. However, in Korea, most of the vaccines used are standard-dose egg-based vaccines, which might contribute to the lower VE observed. Because seasonal influenza epidemics can persist for more than six months, it is essential to consider introducing highly immunogenic influenza vaccines into the National Immunization Program to enhance long-term preventive efficacy.

This study has some limitations. There are currently no available national laboratory surveillance data for the 2023–2024 influenza season; therefore, we cannot determine whether the vaccine strain matched the circulating strain. Considering the differences in VE across regions, laboratory surveillance data are essential for analyzing the results and selecting future vaccine strains. Finally, due to the insufficient study sample size, it was challenging to confirm statistically significant preventive effects of the influenza vaccine against severe disease, such as influenza-related hospitalizations and ICU admissions.

In conclusion, this study confirmed the influenza VE (24%) for the 2023–2024 season in Korea. Notably, the lower preventive effectiveness observed in older adults highlights the need to consider introducing highly immunogenic vaccines.

## Figures and Tables

**Table 1:** Demographic and clinical characteristics of the study participants.

	No. of Participants (n = 3390)	Test-Positive Cases (n = 1695)	Test-Negative Controls (n = 1695)	*p*-Value
Month of enrollment				1.000
November, 2023	550 (16.2)	275 (16.2)	275 (16.2)	
December, 2023	1590 (47.0)	795 (47.0)	795 (47.0)	
January, 2024	984 (29.0)	492 (29.0)	492 (29.0)	
February, 2024	208 (6.1)	104 (6.1)	104 (6.1)	
March, 2024	42 (1.2)	21 (1.2)	21 (1.2)	
April, 2024	16 (0.0)	8 (0.0)	8 (0.0)	
Age group				1.000
19–49 years	1988 (58.6)	994 (58.6)	994 (58.6)	
50–64 years	532 (15.7)	266 (15.7)	266 (15.7)	
≥65 years	870 (25.7)	435 (25.7)	435 (25.7)	
Sex				0.187
Female	1950 (57.5)	994 (58.6)	956 (56.4)	
Male	1440 (42.5)	701 (41.4)	739 (43.6)	
Comorbidities				
None	2206 (65.1)	1145 (67.6)	1061 (62.6)	0.002 *
Diabetes mellitus	450 (13.3)	222 (13.1)	228 (13.5)	0.761
Cardiovascular disease	233 (6.9)	109 (6.4)	124 (7.3)	0.309
Chronic lung disease	207 (6.1)	97 (5.7)	110 (6.5)	0.351
Chronic renal disease	142 (4.2)	58 (3.4)	84 (5.0)	0.032 *
Chronic liver disease	61 (1.8)	21 (1.2)	40 (2.4)	0.014 *
Chronic neurological disease	269 (7.9)	130 (7.7)	139 (8.2)	0.567
Solid malignancy	238 (7.0)	86 (5.1)	152 (9.0)	<0.001 *
Hematologic malignancy	37 (1.1)	19 (1.1)	18 (1.1)	0.869
Immunosuppressant agent use	100 (2.9)	34 (2.0)	66 (3.9)	0.001 *
HIV	5 (0.1)	1 (0.1)	2 (0.2)	0.179
Influenza vaccination, 2023/2024 season	1294 (38.2)	610 (36.0)	684 (40.4)	0.009 *
Admission	963 (28.4)	387 (22.8)	576 (34.0)	<0.001 *
ICU admission	156 (4.6)	61 (3.6)	95 (5.6)	<0.001 *
Mortality	57 (1.7)	22 (1.3)	35 (2.1)	<0.001 *

Data are presented as the number (%). HIV, human immunodeficiency virus; ICU, intensive care unit. * *p* < 0.05.

**Table 2:** Influenza vaccine effectiveness against influenza-related hospitalization and ICU admission.

	Test-Positive, Vaccinated/Total (%)	Test-Negative, Vaccinated/Total (%)	Adjusted VE (95% CI) (%)	*p*-Value
Hospitalization	204/387 (52.7)	269/576 (46.7)	16.5 (−13.9 to 38.8)	0.254
ICU admission	25/61 (41.0)	50/95 (52.6)	55.2 (−0.2 to 80.0)	0.051

VE, vaccine effectiveness; CI, confidence interval; ICU, intensive care unit.

**Table 3:** Comparison of vaccine effectiveness between the late and early periods of the influenza season.

	Test-Positive, Vaccinated/Total (%)	Test-Negative, Vaccinated/Total (%)	Adjusted VE (95% CI) (%)	*p*-Value
Early period (November to December, 2023)				
Overall	377/1070 (35.2)	415/1070 (38.8)	21.5 (4.3 to 35.5)	0.016 *
Influenza A	371/1036 (35.8)	415/1070 (38.8)	20.4 (2.9 to 34.8)	0.025 *
Late period (January to April, 2024)				
Overall	233/625 (37.3)	269/625 (43.0)	28.1 (7.2 to 44.4)	0.011 *
Influenza A	208/476 (43.7)	269/625 (43.0)	12.4 (−14.9 to 33.2)	0.339

VE, vaccine effectiveness; CI, confidence interval. * *p* < 0.05.

## Data Availability

All data have been presented in this article and Supplementary Materials.

## Supplementary Materials

The following supporting information can be downloaded at: https://www.mdpi.com/article/10.3390/vaccines13020197/s1, Table S1: Comparison of RAT and RT-PCR results; Table S2: Estimated influenza vaccine effectiveness based on influenza subtypes; Table S3: Influenza test results in the late and early periods of the season.
