# Supplementary material for: Early and Late Influenza Vaccine Effectiveness in South Korea During the 2023–2024 Season
Source: Vaccines (Basel). 2025 Feb 17;13(2):197. doi: 10.3390/vaccines13020197 (PMC11861641; doi:10.3390/vaccines13020197)
Supplement: Supplementary file 1 [file vaccines-13-00197-s001.zip › vaccines-3423336-supplementary.pdf]

Supplementary Table S1. Comparison of RAT and RT-PCR results

RT-PCR	RAT				
	Negative	Influenza A	Influenza B	Not done	Total
Negative	173	11	0	45	229
Influenza A	52	66	0	52*	170
Influenza B	3	0	3	4*	10
Not done	1476	1331	174	0	2981
Total	1704	1408	177	101	3390

RAT, rapid antigen testing; RT-PCR, reverse transcription polymerase chain reaction

Matching RAT and RT-PCR results are indicated in gray color.

* There is one case where RAT is not available and A and B appear simultaneously in RT-PCR tests

Supplementary Table S2. Estimated influenza vaccine effectiveness based on influenza subtypes

	Test-positive, vaccinated/total (%)	Test-negative, vaccinated/total (%)	Adjusted VE (95% CI) (%)	P-value
**Influenza**				
Overall	610/1695 (36.0)	684/1695 (40.4)	24.3 (11.5 to 35.2)	< 0.001*
19–49 years	239/994 (24.0)	305/994 (30.7)	31.1 (15.7 to 43.7)	< 0.001*
50–64 years	56/266 (21.1)	60/266 (22.6)	10.9 (-36.3 to 41.8)	0.594
≥ 65 years	315/435 (72.4)	319/435 (73.3)	13.5 (-17.9 to 36.6)	0.358
**Influenza A**				
Overall	580/1512 (38.4)	684/1695 (40.4)	19.6 (5.7 to 31.4)	0.007*
19–49 years	217/836 (26.0)	305/994 (30.7)	23.8 (6.1 to 38.2)	0.011*
50–64 years	53/249 (21.3)	60/266 (22.6)	12.2 (-35.3 to 43.0)	0.556
≥ 65 years	310/427 (72.6)	319/435 (73.3)	13.1 (-18.7 to 36.3)	0.378
**Influenza A/H1N1**				
Overall	36/79 (45.6)	684/1695 (40.4)	36.5 (-8.9 to 63.0)	0.099
19–49 years	0/5 (0.0)	305/994 (30.7)	N/A	N/A
50–64 years	5/18 (27.8)	60/266 (22.6)	-86.8 (-506.5 to 42.5)	0.298
≥ 65 years	31/56 (55.4)	319/435 (73.3)	46.9 (2.5 to 71.1)	0.041*
**Influenza A/H3N2**				
Overall	48/86 (44.6)	684/1695 (40.4)	13.2 (-97.4 to 20.8)	0.590
19–49 years	2/12 (16.7)	305/994 (30.7)	49.5 (-139.0 to 89.3)	0.389
50–64 years	0/15 (0.0)	60/266 (22.6)	N/A	N/A
≥ 65 years	46/59 (78.0)	319/435 (73.3)	-38.6 (-173.4 to 29.8)	0.347
**Influenza B**				
Overall	31/184 (16.0)	684/1695 (40.4)	60.1 (39.4 to 73.8)	< 0.001*
19–49 years	22/158 (13.9)	305/994 (30.7)	64.8 (43.5 to 78.1)	< 0.001*
50–64 years	3/17 (17.6)	60/266 (22.6)	27.0 (-178.9 to 80.9)	0.645
≥ 65 years	6/9 (66.7)	319/435 (73.3)	40.3 (-185.8 to 87.5)	0.519

VE, vaccine effectiveness; CI, confidence interval; N/A, not available

* $P < 0.05$

Supplementary Table S3. Influenza test results in the late and early periods of the season

Influenza subtypes	Early period (November to December, 2023) (n = 1,070)	Late period (January to April, 2024) (n = 625)	Total (N = 1,695)
Influenza A	1,036 (96.8)	476 (76.2)	1,512 (89.2)
A/H1N1*	72 (62.6)	7 (14.0)	79 (47.9)
A/H3N2*	43 (37.4)	43 (86.0)	86 (52.1)
Influenza B	35 (3.3)	149 (23.8)	184 (10.8)

Data are presented as the number (%). During the early period, a patient was positive for both influenza A and B.

* The proportions were calculated only for cases where subtyping was possible through polymerase chain reaction testing.
